# ‘Functional Connectivity’ Is a Sensitive Predictor of Epilepsy Diagnosis after the First Seizure

**DOI:** 10.1371/journal.pone.0010839

**Published:** 2010-05-26

**Authors:** Linda Douw, Marjolein de Groot, Edwin van Dellen, Jan J. Heimans, Hanneke E. Ronner, Cornelis J. Stam, Jaap C. Reijneveld

**Affiliations:** 1 Department of Neurology, VU University Medical Center, Amsterdam, The Netherlands; 2 Department of Clinical Neurophysiology, VU University Medical Center, Amsterdam, The Netherlands; Indiana University, United States of America

## Abstract

**Background:**

Although epilepsy affects almost 1% of the world population, diagnosis of this debilitating disease is still difficult. The EEG is an important tool for epilepsy diagnosis and classification, but the sensitivity of interictal epileptiform discharges (IEDs) on the first EEG is only 30–50%. Here we investigate whether using ‘functional connectivity’ can improve the diagnostic sensitivity of the first interictal EEG in the diagnosis of epilepsy.

**Methodology/Principal Findings:**

Patients were selected from a database with 390 standard EEGs of patients after a first suspected seizure. Patients who were later diagnosed with epilepsy (i.e. ≥two seizures) were compared to matched non-epilepsy patients (with a minimum follow-up of one year). The synchronization likelihood (SL) was used as an index of functional connectivity of the EEG, and average SL per patient was calculated in seven frequency bands. In total, 114 patients were selected. Fifty-seven patients were diagnosed with epilepsy (20 had IEDs on their EEG) and 57 matched patients had other diagnoses. Epilepsy patients had significantly higher SL in the theta band than non-epilepsy patients. Furthermore, theta band SL proved to be a significant predictor of a diagnosis of epilepsy. When only those epilepsy patients without IEDs were considered (n = 74), theta band SL could predict diagnosis with specificity of 76% and sensitivity of 62%.

**Conclusion/Significance:**

Theta band functional connectivity may be a useful diagnostic tool in diagnosing epilepsy, especially in those patients who do not show IEDs on their first EEG. Our results indicate that epilepsy diagnosis could be improved by using functional connectivity.

## Introduction

Epilepsy is the most frequently occurring disease of the central nervous system, affecting approximately 1% of the world's population [1]. Despite enormous research efforts, the pathogenesis of epilepsy is not fully understood [2], which hampers both adequate diagnosis as well as subsequent treatment of epilepsy patients. Underdiagnosis and overdiagnosis present important problems for patients, as they are either at risk of having another seizure, or take unnecessary antiepileptic drugs (AEDs) that may have significant side effects.

The clinical diagnosis of epilepsy is based on the criteria of the International League Against Epilepsy (ILAE). Clinical history taking is usually combined with an interictal electro-encephalogram (EEG), on which so-called ‘interictal epileptiform discharges’ (IEDs; certain graphic elements on an EEG recording) may be identified. Unfortunately, while inspection of the first EEG is highly specific as a diagnostic tool, it is not very sensitive: approximately 30 to 50% of epilepsy patients actually have IEDs on their first EEG [3]. This percentage increases with repeated EEG recordings, but between 2 and 18% of patients never have IEDs on their EEGs [4], [5]. Also, approximately 0·5% of the healthy population display IEDs [6], [7]. Thus, the development of an EEG measure that is more sensitive than IEDs, whilst preserving high specificity, would be highly valuable in diagnosis and treatment of epilepsy.

A relatively new concept in neuroscience is ‘functional connectivity’. This notion refers to the statistical interdependencies (or synchronization) between time series from different brain areas, as measured by EEG, magnetoencephalography (MEG), or functional magnetic resonance imaging (fMRI). Synchronization of neurons may be pivotal for optimal brain functioning [8], but it can also reflect abnormal dynamics related to epilepsy. Several studies indicate that changes in synchronization occur before and during the seizure [9], [10], [11], [12], [13]. Interictally, increased EEG and depth electrode synchronization during the seizure in patients with medial temporal lobe epilepsy has been reported previously [14], [15]. When comparing patients with healthy control subjects, increased EEG synchronization in particularly the delta and beta bands was found in long-term epilepsy patients [16]. Thus, global synchronization differs between epilepsy patients and healthy subjects in the interictal state, but these changes might already be present in the early stages of the disease. If so, determination of functional connectivity may aid in the diagnosis of epilepsy. Indeed, children with absence seizures could be differentiated from healthy children by application of connectivity in their interictal EEGs [17], as were children suffering from mixed types of idiopathic epilepsy [18]. The current study investigates functional connectivity of the first EEG of adult patients with suspected epileptic seizures, since sensitivity of the first EEG is currently insufficient. Functional connectivity of the first EEG after an initial suspected seizure is explored as a diagnostic tool for epilepsy.

## Methods

### Ethics Statement

All data used in this study were collected as part of standard medical care and were analyzed anonymously. Approval from the medical ethics committee of the VU University Medical Center was obtained, which agreed no informed consent was needed retrospectively.

### Patients

For this retrospective study, the database of EEG recordings performed in the VU University Medical Center between October 1^st^ 2003 and September 1^st^ 2008 was used. From this database, we selected those patients (age >18 years old) who were evaluated with a standard EEG because of suspected epilepsy after a first possible seizure. Medical chart review was conducted for all patients to determine whether a clinical diagnosis of epilepsy was reached within a follow-up of one year. We aimed to form two groups: (1) a group of patients who were diagnosed with epilepsy (defined as two or more epileptic seizures according to the International League Against Epilepsy), with or without IEDs on their EEG, and (2) a group of patients who were initially suspected of having epilepsy, but were not diagnosed as such. Follow-up of at least one year was an inclusion criterion for the latter group, ensuring that no second seizure occurred. This non-epilepsy patient population was individually matched to the patient group with regard to age and sex. Additional clinical data of the included patients were collected from their medical chart when available, including type of epilepsy, imaging reports (computed tomography (CT) or magnetic resonance images (MRI)), and information regarding drug use at the time of the EEG.

### Electroencephalography recordings

EEGs were recorded with a digital EEG apparatus (Brainlab, manufactured by OSG) from Fp2, Fp1, F8, F7, F4, F3, A2, A1, T4, T3, C4, C3, T6, T5, P4, P3, O2, O1, Fz, Cz and Pz with tin electrodes. Impedance was kept below 5 KOhm. Initial filter settings were: time constant 1 s and high frequency cut-off 70 Hz. Sampling frequency was 500 Hz and A–D precision 16 bit. An average reference montage was used.

EEGs of all eligible patients were visually inspected [LD]; only artifact-free epochs were included in this study, with or without IEDs as determined at the time of diagnosis by experienced clinical neurophysiologists. From the EEG of around 30 minutes, four epochs of eight seconds (4096 samples) during resting-state with closed eyes were selected. The two frontoparietal and basal temporal electrodes (Fp1, Fp2, A1, and A2) were excluded to minimize artifacts due to eye movements. The analyses were performed on the remaining 17 electrodes. The selected epochs were converted to ASCII-files, after which functional connectivity was calculated with software available at the department of clinical neurophysiology (DIGEEGXP [CJS]).

### Functional connectivity

The synchronization likelihood (SL [19]) was used as an index of functional connectivity. The SL is based on the concept of generalized synchronization [20], and takes linear as well as nonlinear synchronization between two time series into account (see [21] for lag, embedding dimension, and filtering parameters). SLs between all pairs of electrodes were determined in the following seven frequency bands: delta (0·5–4 Hz), theta (4–8 Hz), lower alpha (8–10 Hz), upper alpha (10–13 Hz), beta (13–30 Hz), lower gamma (30–45 Hz), and upper gamma (55–80 Hz; see [22]). Subsequently, the SL matrix (17×17) was averaged to obtained a mean connectivity value for each patient and each epoch, after which the four epochs per patient were again averaged. This yielded seven SL values (one for each frequency band) for each patient.

### Statistical analysis

All statistical analyses were performed using SPSS 15.0 for Windows. Differences between epilepsy and non-epilepsy patients were investigated using Student's t-tests and Chi-square tests, as were differences between epilepsy patients with and without IEDs on their EEG.

Differences in SL between epilepsy and non-epilepsy patients, and epilepsy patients with or without IEDs were investigated using non-parametric Mann-Whitney exact U-tests, since SL does not follow a normal distribution. P-values were corrected for multiple testing using the Bonferroni method (corrected for seven tests: one for each frequency band).

In order to explore whether SL was able to classify patients correctly with respect to epilepsy diagnosis, logistic regression analysis was performed, which is relatively robust to violations of the normal distribution.

## Results

### Patient characteristics

**Figure 1:**
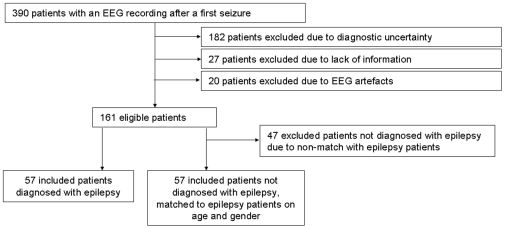
Flowchart of included patients.

The database with EEG recordings because of suspected epilepsy after a first seizure contained 390 patients. Of this group, 57 patients with a definite diagnosis of epilepsy remained after excluding those who did not meet inclusion criteria (see figure 1). A total of 104 participants were not diagnosed with epilepsy, and 57 participants out of this group were individually matched regarding gender and age to the 57 epilepsy patients. All patients were referred to the VU University Medical Center (which is a tertiary referral center and also has a large emergency department) by their general physician or reported themselves at the emergency department of our hospital, after having one episode that could be explained as an epileptic seizure. All diagnoses were finally reached by the staff neurologists in the VU University Medical Center, also making use of the EEG report of the clinical neurophysiologist of this hospital. Causes for the suspected seizure in these patients are listed in table 1; no other diagnosis was reached in three patients, but epilepsy was ruled out as a diagnosis. No significant differences in age or gender were present between the 57 patients who were included and the 47 who were not. None of the patients used AEDs at the time of the first EEG.

**Table 1:** Other diagnoses after first seizure and EEG in non-epileptic patients (n = 57).

	Number of patients (%)
Stress or psychological cause	17 (29)
Vasovagal collapse	7 (12)
Cardial disturbance	6 (11)
Transient ischemic attack	5 (9)
Brain contusion	4 (7)
Neuropathy	3 (5)
Sleeping disorders	3 (5)
Hypoglycemia	3 (5)
Migraine	2 (4)
Drug abuse	2 (4)
Motor neuron disease	1 (2)
Orthostatic hypotension	1 (2)
No diagnosis reached	3 (5)

Some of the patients were found to have intracranial abnormalities on CT or MRI (see table 2). This was the case in 24 epilepsy patients and in 14 patients who were not diagnosed with epilepsy. These abnormalities were not necessarily related to the possible seizures according to the radiologist and/or clinical neurophysiologist. Some of the patients used medication influencing the central nervous system (CNS; see table 2). Thirteen of the non-epilepsy patients used CNS medication, while this was the case in 12 epilepsy patiens. Of the 57 patients suffering from epilepsy, 20 had IEDs on their EEG (at the time of diagnosis, determined by experienced clinical neurophysiologists), while none of the non-epilepsy patients did. Since patients were individually matched, there were no significant differences in age or sex between epilepsy and non-epilepsy patients, nor did they differ regarding radiological abnormalities or the use of medication influencing the CNS. Epilepsy patients with IEDs on their EEG did not differ from epilepsy patients without IEDs with regard to abovementioned variables.

**Table 2:** Patient characteristics (n = 114).

	Non-epileptic patients (n = 57)		Epileptic patients (n = 57)	
		Total (n = 57)	No IEDs (n = 37)	IEDs (n = 20)
Age in years (SD)	54 (17)	50 (18)	48 (19)	53 (17)
Sex: male (%)	28 (49)	28 (49)	22 (59)	6 (30)
IEDs on EEG (%)**	-	20 (35)	-	20 (35)
Type of epilepsy				
Partial (%)	-	21 (37)	12 (32)	9 (45)
Generalized (%)	-	36 (63)	25 (68)	11 (55)
Radiological abnormalities				
No abnormalities (%)	30 (53)	32 (55)	22 (59)	10 (50)
White matter abnormalities (%)	8 (14)	7 (11)	3 (8)	4 (20)
Meningioma (%)	2 (4)	2 (4)	2 (5)	-
Low-grade astrocytoma (%)	-	2 (4)	2 (5)	-
Glioblastoma multiforme (%)	-	2 (4)	1 (3)	1 (5)
Pituitary gland tumor (%)	1 (2)	-	-	-
Brain metastasis (%)	-	2 (4)	1 (3)	1 (5)
Cortical atrophy (%)	1 (2)	4 (7)	1 (3)	3 (15)
Arachnoidal cyste (%)	-	2 (4)	1 (3)	1 (5)
Other	2 (4)	3 (5)	3 (8)	-
No imaging available	13 (23)	1 (2)	1 (3)	-
CNS medication				
Sedatives (%)	4 (7)	4 (7)	3 (8)	1 (5)
Antidepressants (%)	8 (14)	4 (7)	3 (8)	1 (5)
Anti-migraine (%)	1 (2)	-	-	-
Corticosteroids (%)	-	2 (4)	1 (3)	1 (5)
Antiepileptic drugs (%)	-	1 (2)	-	1 (5)
Cholinesterase inhibitor (%)	-	1 (2)	-	1 (5)

Note. ** significant difference (p<.001) between epileptic and non-epileptic patients of total group (n = 114), IEDs = interictal epileptiform discharges, CNS = central nervous system.

### SL differences

**Figure 2:**
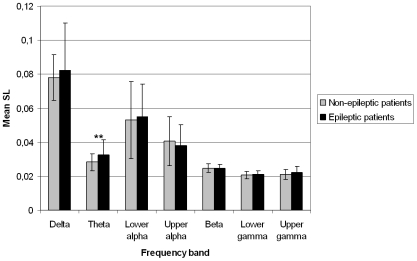
Mean SLs of epilepsy and non-epilepsy patients in all seven frequency bands. Note. ** significant difference between patients and controls, p<.001.

Significant differences in functional connectivity were present between epilepsy and non-epilepsy patients in the theta band (see figure 2). In this frequency band, epilepsy patients had significantly higher SL (M = 0.033, SD = 0.009) than non-epilepsy patients (M = 0.028, SD = 0.005; *U* = 1047, *p*<.001). Connectivity in other frequency bands did not differ significantly between groups. There were no significant differences in SL between patients with and without IEDs or patients with partial or generalized seizures.

Some patients had radiological abnormalities (38 patients) and/or used medication that could influence the CNS (25 patients). In order to investigate whether these variables had an impact on the reported differences, we tested SL between patients with and without radiological abnormalities, and with and without CNS medication. Whether or not patients had radiological abnormalities did not influence SL significantly in any frequency band. However, patients using CNS medication had significantly lower upper alpha band SL (M = 0.036, SD = 0.011) than those who did not use such drugs (M = 0.040, SD = 0.014; U = 818, p = .044), as well as lower beta band SL (with M = 0.024, SD = 0.002; without M = 0.025, SD = 0.003; U = 746, p = .012).

Power was also analysed using Fast Fourier Transformations. Patients with epilepsy had significantly higher theta band power than the patients without epilepsy (*U* = 879, *p*<.001)

### SL as predictor of diagnosis

In order to explore whether SL was a useful tool to classify individual patients in the epilepsy or non-epilepsy group, we performed logistic regression with diagnosis (epilepsy versus no epilepsy) as the dependent variable. First, the presence of IEDs on the EEG was used as a predictor of status. Specificity of this model was 100%, while sensitivity was only 35%. The total accuracy was 67%, and the model was a significant predictor of diagnosis (chi-square = 32.0, p<.001).

**Figure 3:**
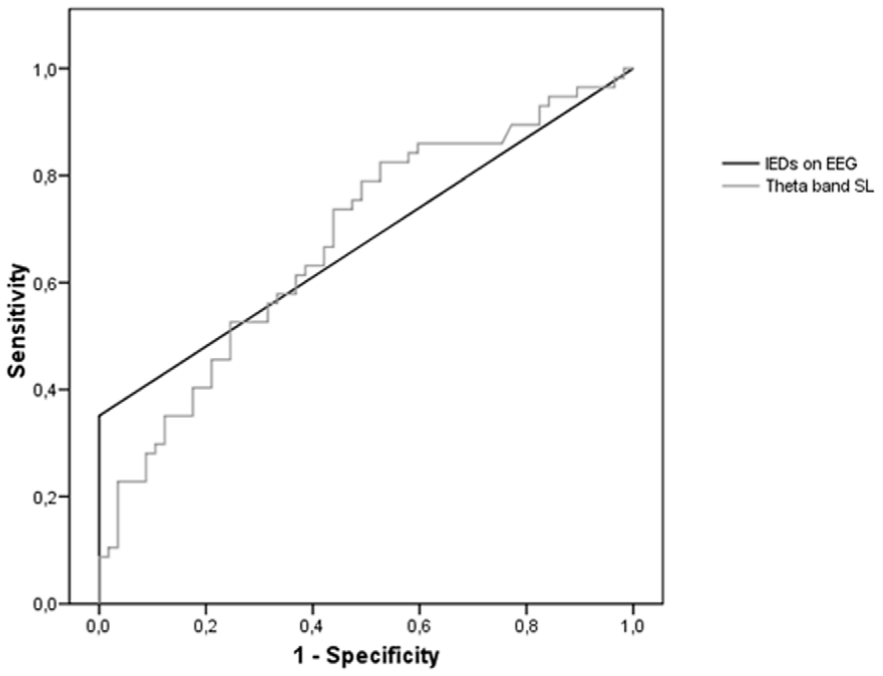
ROC curve of IEDs and theta band SL as predictors of diagnosis in all patients (n = 114).

Subsequently, we added theta band SL to the regression analysis (using backward L-R analysis). This model was significant (chi-square = 43.6, p<.001), and theta band SL was a significant predictor (Exp(B) = 2.38, p = .003). The high value of the beta coefficient indicates that lower theta band SL decreases the odds of being diagnosed with epilepsy, corroborating the abovementioned difference between epilepsy and non-epilepsy patients. The addition of theta band SL to the model yielded overall accuracy of 75% and specificity of 91%, while sensitivity went up to 58%. When only using theta band SL as a predictor of status, the significant model (chi-square = 11.3, p<.001) was accurate in 61% of cases, with specificity of 70% and sensitivity of 53%. The predictive power of theta band SL can also be observed in the ROC curve (see figure 3). Because some patients had radiological abnormalities and/or used medication that could influence these results, both confounders were entered as variables in the logistic regression (using backward analysis). However, radiological abnormalities and CNS-medication were not significant predictors of diagnosis and were removed from the model.

**Figure 4:**
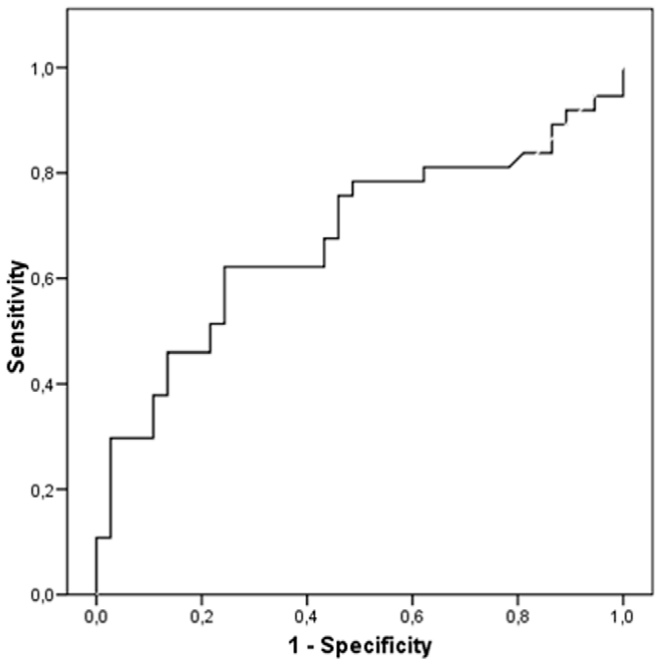
ROC curve of theta band SL as predictor of diagnosis in epilepsy patients without IEDs and their matched non-epilepsy patients (n = 74).

Predictive significance of connectivity would be even more interesting in those patients without IEDs, as no information from the EEG can be used in this population up till now. Therefore, we performed logistic regression analysis on epilepsy patients without IEDs on their EEGs and their matched non-epilepsy patients only (n = 74, see table 3 for patient characteristics). This model was significant (chi-square = 8.0, p = .005), as was theta band SL as a predictor (Exp(B) = 1.86, p = .015). Theta band SL accurately classified 69% of cases; specificity was 76%, while sensitivity was 62% (see figure 4 for ROC curve). When adding radiological abnormalities and medication use to the regression analysis, these two variables were again removed from the model, while theta band SL remained a significant predictor.

**Table 3:** Patient characteristics epileptic patients without IEDs and matched non-epileptic patients (n = 74).

	Non-epileptic patients (n = 37)	Epileptic patients (n = 37)
Age in years (SD)	49 (17)	48 (19)
Sex: male (%)	22 (59)	22 (59)
Type of epilepsy		
Partial (%)	-	12 (32)
Generalized (%)	-	25 (68)
Radiological abnormalities		
No abnormalities (%)	25 (68)	22 (60)
White matter abnormalities (%)	3 (8)	3 (8)
Meningioma (%)	-	2 (5)
Low-grade astrocytoma (%)	-	2 (5)
Glioblastoma multiforme (%)	-	1 (3)
Pituitary gland tumor (%)	1 (3)	-
Brain metastasis (%)	-	1 (3)
Cortical atrophy (%)	-	1 (3)
Arachnoidal cyste (%)	-	1 (3)
Other	2 (5)	3 (8)
No imaging available	6 (16)	1 (3)
CNS medication		
Sedatives (%)	3 (8)	3 (8)
Antidepressants (%)	4 (11)	3 (8)
Anti-migraine (%)	1 (3)	-
Corticosteroids (%)	-	1 (3)

Note. IEDs = interictal epileptiform discharges, CNS = central nervous system.

The predictive value of theta band power was explored, which also yielded significant results. Theta power was a significant predictor of diagnosis in the whole group (chi-square = 17.1, p<.001, with specificity of 77% and sensitivity of 58%) and in the subgroup of patients without IEDs (chi-square = 9.4, p = .002, with specificity of 73% and sensitivity of 51%). These results show that although theta power is also a significant predictor of diagnosis, theta band SL yields higher accuracy.

## Discussion

Differences in EEG functional connectivity between epilepsy and non-epilepsy patients after a first suspected seizure were found: patients diagnosed with epilepsy showed increased synchronization likelihood (SL) in the theta band when compared to patients who were not diagnosed with epilepsy. More importantly, theta band SL on the first EEG proved to be a significant predictor of the diagnosis ‘epilepsy’. Adding theta band SL to IEDs as predictors decreased specificity from 100 to 91%, but sensitivity rose from 35 to 58%. In the group of patients without IEDs, theta band SL as a predictor had specificity of 76%, while sensitivity was 62%. These results indicate that functional connectivity may be a powerful tool providing support for the diagnosis of epilepsy, especially in those patients who do not show IEDs on their first EEG.

Epilepsy is characterized by changes in functional connectivity of the brain: much research has focused on changes in connectivity during the seizure. In the interictal period, increased synchronization in the EEG and in depth electrodes has been reported previously [14], [15]. Furthermore, higher delta and beta synchronization has been reported in long-term epilepsy patients who were on antiepileptic medication when compared to healthy subjects [16]. The current results corroborate these studies and suggest that interictal brain connectivity of epilepsy patients deviates from patients without epilepsy. Increased low-frequency connectivity has also been reported in other brain diseases. Brain tumor patients (who often suffer from epilepsy) display a pathological increase of theta band synchronization when compared to healthy controls [23], [24], as do Alzheimer's patients [22], and Parkinson's patients [25]. Several hypotheses have been formulated regarding these findings: the increased synchronization in the theta band may reflect a compensatory mechanism, but it may also be a display of synchronization disinhibition as a consequence of brain disease. Furthermore, the increased connectivity may be caused by abnormal plasticity (i.e. an outgrowth of many connections) after a lesion [26]. In the current study, no healthy controls were included, which makes inferences of causes for increased theta band connectivity difficult.

At present, diagnosis of epilepsy is mainly based on clinical judgment, but there is a need for reliable diagnostic tools to aid diagnosis and classification of epilepsy syndromes and therapeutic decisions. In EEGs recorded for the (differential) diagnosis of epilepsy, the presence of interictal epileptiform discharges (IEDs) is an important feature. Patients with one suspected seizure and IEDs on their EEG are to be treated with antiepileptic medication according to current guidelines of the ILAE. However, only 30–50% of patients actually have IEDs on their first EEG [3]. In previous research, SL has been used to distinguish between sleep terrors and nocturnal frontal lobe epilepsy seizures [27]. The SL proved able to detect seizures while disregarding parasomnias associated with sleep terrors. Measures of functional connectivity have been investigated with respect to their ability to predict seizures [28], but these methods are only applied to patients already diagnosed with epilepsy. Previous studies used functional connectivity to differentiate between children with epileptic seizures and healthy children based on their resting-state EEG [17], [18]. To our knowledge, functional connectivity has never been used as a method of differentiating between new adult epilepsy patients and patients with a first seizure who are later not diagnosed with epilepsy.

The current study has some limitations. The actual predictive power of functional connectivity when diagnosing epilepsy can only be proven in prospective studies, whereas the current patient data were acquired retrospectively. This also limited our sample size, since strict criteria were applied to determine whether patients were diagnosed with epilepsy or not. In addition, in-depth analysis of variables such as epilepsy type was impossible because of missing data. Also, participants were heterogeneous with respect to radiological abnormalities and CNS medication use. Prospective studies with more homogeneous patients and elaborate data collection are needed to confirm our findings. Furthermore, results of power analysis show that theta band power was also a significant predictor of diagnosis. This is not surprising, as synchronization likelihood is sensitive to volume conduction and is closely related to power. However, power performed poorer than theta band functional connectivity in terms of specificity and sensitivity in our regression models, indicating the added value of connectivity over power. Moreover, the commonly held idea that connectivity differences are a result of power alterations may not be correct. It is possible that the opposite is true, namely that changes in connectivity may induce power changes. Future studies may address this association.

If connectivity could be used as a predictive tool in this patient group, this would imply great benefits in clinical practice. Time and resources would be saved when patients do not have IEDs on their EEG and would in the current situation undergo a second EEG after sleep deprivation, which is still not very sensitive. First and foremost, however, correct diagnosis directly after a first seizure would mean great health benefit. At this time, many patients cannot be diagnosed with epilepsy until they experience a second seizure or have IEDs on their first or second EEG. If correct diagnosis could be reached earlier and antiepileptic drugs could be prescribed immediately, this would minimize the risk of epilepsy-related accidents. Furthermore, better diagnosis would result in less unnecessary AED use in patients who do not have epilepsy. In conclusion, functional connectivity is a promising new tool to diagnose epilepsy, especially in those patients who have a normal first EEG.
